# Developing dual herbicide tolerant transgenic rice plants for sustainable weed management

**DOI:** 10.1038/s41598-018-29554-9

**Published:** 2018-08-02

**Authors:** Dhirendra Fartyal, Aakrati Agarwal, Donald James, Bhabesh Borphukan, Babu Ram, Vijay Sheri, Pawan K. Agrawal, V. Mohan Murali Achary, M. K. Reddy

**Affiliations:** 1 Crop Improvement Group, International Centre for Genetic Engineering and Biotechnology, Aruna Asaf Ali Marg, New Delhi, 110067 India; 2 Uttarakhand Technical University, Dehradun, Uttarakhand India; 3 Plant Molecular Biology Lab, Department of Botany, University of Delhi, New Delhi, India; 4 National Agricultural Science Fund, Indian Council of Agricultural Research, New Delhi, India

## Abstract

Herbicides are important constituents of modern integrated weed management system. However, the continuous use of a single herbicide leads to the frequent evolution of resistant weeds which further challenges their management. To overcome this situation, alternating use of multiple herbicides along with conventional weed-management practices is suitable and recommended. The development of multiple herbicide-tolerant crops is still in its infancy, and only a few crops with herbicide tolerance traits have been reported and commercialized. In this study, we developed transgenic rice plants that were tolerant to both bensulfuron methyl (BM) and glufosinate herbicides. The herbicide tolerant mutant variant of rice *AHAS* (*Acetohydroxyacid synthase*) was overexpressed along with codon optimized bacterial *bar* gene. The developed transgenic lines showed significant tolerance to both herbicides at various stages of plant development. The selected transgenic lines displayed an increased tolerance against 100 μM BM and 30 mg/L phosphinothricin during seed germination stage. Foliar applications further confirmed the dual tolerance to 300 μM BM and 2% basta herbicides without any significant growth and yield penalties. The development of dual-herbicide-tolerant transgenic plants adds further information to the knowledge of crop herbicide tolerance for sustainable weed management in modern agricultural system.

## Introduction

The presence of weeds is very serious problem in any agricultural system because they aggressively compete with crops for nutrients, light and other important resources. Their infestation imposes a severe challenge to crop yield, productivity and survival of plants. Among various conventional and modern strategies, chemical herbicides are the least expensive, easiest and most effective way to combat weed invasion^1–3^. Advancements in genetically modified herbicide resistance technology, which began with the introduction of the first glyphosate-resistant soybean variety in 1996, opened a new way to manage weed populations in crop fields. Since then, many important genetically modified crops that are tolerant to various herbicides have been developed and commercialized^4,5^. Most of the transgenic crops are transformed with single herbicide-tolerance genes against glyphosate, basta, HPPD, PPO or AHAS-inhibiting herbicides. With the rise in global demand for food and other agricultural products, herbicide technology has contributed greatly during the past few decades. However, the continuous overuse of a single herbicide multiple times in a growing season increases the potential risk of evolution of resistant weeds which has become a major concern in agriculture worldwide. Further, higher doses of herbicide applications cause major threats to agricultural land and the environment^6,7^. Many weeds which are resistant to various herbicides have been identified in different parts of the world^1,7–9^. According to the ‘International Survey of Herbicide Resistant Weeds’, 486 unique cases of herbicide-resistant weed biotypes have been reported globally, of which 147 are in dicot and 106 are in monocot groups. A total of 92 crops have been reported to be infested by herbicide-resistant weeds. Glyphosate, basta, AHAS inhibitor, HPPD inhibitor and PPO inhibitor herbicides are widely used to kill or suppress weeds in crop fields. Among these herbicides, the highest 159 identified unique cases of weed resistance were of AHAS-inhibiting herbicides; in addition, there have been 39 cases of resistance identified against glyphosate, 3 against basta, 2 against HPPD-inhibitors and 13 against PPO-inhibiting herbicides, and the number is increasing daily^10^.

To counter the problem of the evolution of resistant weeds and to minimize the overuse of herbicides, alternative agricultural strategies including the use of multiple herbicides in conjunction with traditional methods are suggested which constitutes an effective integrated weed management practice. Moreover, it is extremely important to maintain the diversity in herbicide use for efficiently exploiting the properties of important presently available herbicides to manage the weeds proficiently for the long term. The development of transgenic crops that are resistant to two or more herbicides remains a major challenge and a priority area of agricultural programs. However, the agribiotech or agrichemical companies like Monsanto, Pioneer, Dow, Bayer and Syngenta have proposed or it is in their agenda to introduce multiple herbicide tolerant traits in crops particularly in soybean in addition to glyphosate tolerance trait^11^. Thus, realizing its importance in food security, we report the development of dual-herbicide tolerant transgenic rice plants that exhibit tolerance to basta and BM for sustainable weed management.

AHAS is the first enzyme in the branched-chain amino acid biosynthesis pathway and is the target of many commercially used herbicides including sulfonyl ureas and other related derivatives^12–14^. The AHAS enzyme catalyzes the formation of acetolactate from two molecules of pyruvate which is a precursor for the synthesis of the amino acids valine, leucine, and isoleucine. Many commercially important AHAS inhibitors such as sulfonylureas (SUs), imidazolinones (IMIs), triazolopyrimidines (TPs), pyrimidinyl oxybenzoates (POBs), and sulfonylamino carbonyl triazolinones (SCTs) are marketed and used for pre-emergent or post-emergent applications. The mutant variants of AHAS can tolerate applications of different kinds of herbicides and has been used in many commercially important transgenic crops. Apart from generating transgenic crops tolerant to various AHAS-inhibitor herbicides, scientists have also been able to develop the same with the help of conventional breeding methods which are referred as non-transgenic crops. The development of Clearfield* crops with the help of conventional breeding is the best example of commercially available non-transgenic crops. These crops are tolerant to various kinds of imidazolinone herbicides^15^. Recently, Shobha and colleagues have developed and characterized a novel imidazolinone tolerant mutant in indica rice with the help of EMS technique. These plants were able to tolerate increased application of Imazethapyr herbicide^16^. Piao and colleagues have also developed an imidazolinone resistant japonica rice variety by introducing a resistant form of *AHAS* from indica variety^17^. In addition, there are many reports exploiting the use of different AHAS mutations to develop AHAS-inhibitor tolerant rice and other crops (briefly described in discussion part).

Among the various herbicide tolerant mutations reported in AHAS, the position P197 (with respect to *Arabidopsis*) is highly vulnerable to amino acid substitutions resulting in tolerance against sulfonylurea herbicides. A total of 11 different amino acid substitutions are reported at this position in many tolerant weed biotypes that show a high degree of resistance against several AHAS-inhibiting herbicides^1^.

Similarly, bialaphos is an important non-selective broad-spectrum herbicide that selectively inhibits glutamine synthetase (GS), a key enzyme in the nitrogen assimilation pathway. The *Bar* (Bialaphos resistance) encoding gene detoxifies bialaphos (becomes active after the removal of 2 alanine residues, which is then called phosphinothricin) via the acetylation of its amino group. Many commercially important bialaphos-resistant crops such as canola, corn, cotton and soybean have been developed by introducing *bar* genes from *Streptomyces*; these transgenic crops are successfully cultivated in many parts of the world^18–21^. Here, we developed the dual herbicide-tolerant transgenic plants in popular indica rice cultivar Swarna via the overexpression of rice mutant *OsmAHAS* gene along with the *bar* gene and confirmed their tolerance against BM and basta herbicides at various developmental stages.

## Results

### *In silico* analysis and identification of P197S tolerant mutation in rice AHAS

**Figure 1:**
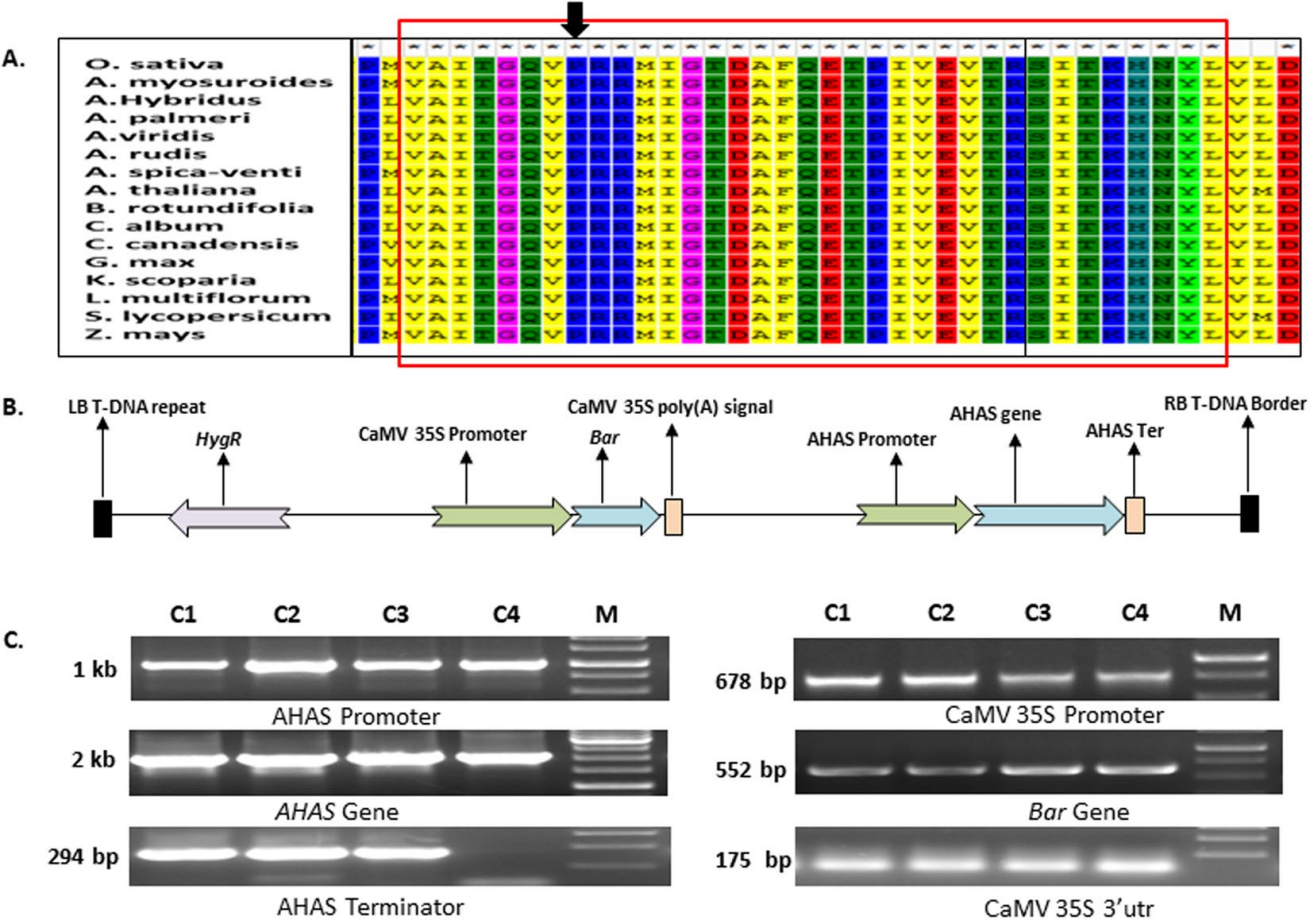
*In silico* analysis of AHAS proteins and the schematic representation and PCR confirmation of the expression cassette. (**A**) Multiple sequence alignment of AHAS proteins in various plant species. The rectangle shows the presence of a 35 amino acid long conserved stretch, and the arrow shows the position of the conserved proline amino acid at position 171. (**B**) Schematic representation of expression cassette containing mutated rice *AHAS* and *bar* genes. (**C**) PCR confirmation of the expression cassettes (Full images are available in Supplementary information as Figs SI3 and 4).

A multiple sequence alignment involving the AHAS enzyme of different plants resulted in a consecutive 35 amino acid long conserved stretch from 190–224 amino acid positions in all AHAS enzymes (Fig. 1A). The amino acid proline was highly conserved in all the tested plant species at 197 with reference to *Arabidopsis*; this residue was located at position 171 in the rice AHAS protein. There have been many AHAS-inhibiting herbicide-resistant weeds with the P197S substitution mutation reported to exist in nature^10^ and the multiple sequence alignment revealed the conserved position of this amino acid in the protein sequence, confirming the crucial role of proline in AHAS enzyme function. Thus, a naturally selected tolerance mutation in the *AHAS* gene could be a valuable resource for the development of herbicide-tolerant transgenic plants.

### Development and molecular confirmation of transgenic plants

**Figure 2:**
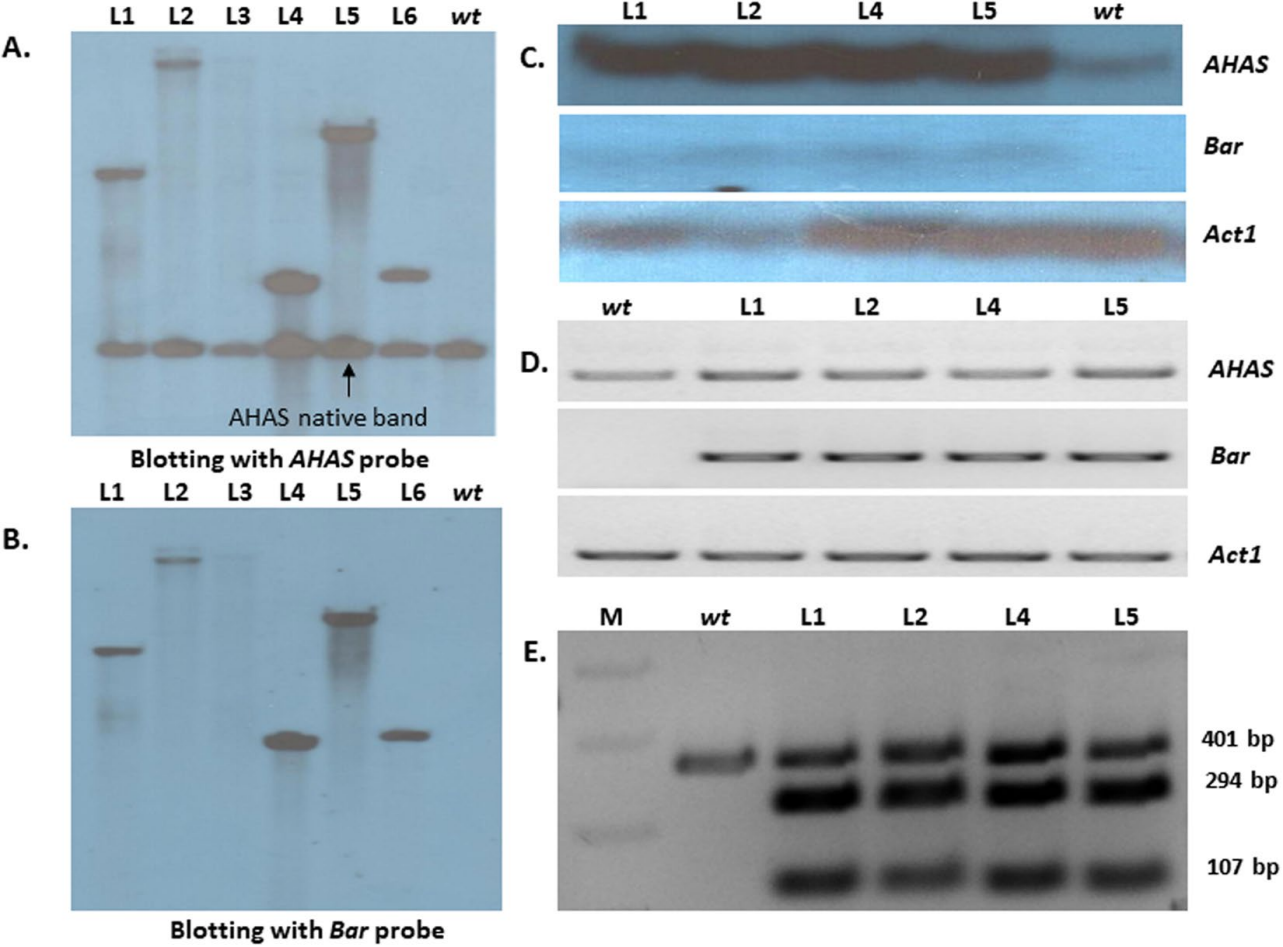
Molecular analysis of transgenic plants. (**A**) and (**B**) Transgene integration analysis of PCR positive transgenic plants by southern blotting. First, the membrane was hybridized with *AHAS* probe showing the presence of common native and unique transgene bands (**A**). The membrane was rehybridized with *bar* probe which shows only a single unique transgene integration (**B**). (**C**) Expression analysis of transgenes by northern blotting. To see the expression of the *AHAS* and *bar* genes, the same membrane was hybridized at first with *AHAS* and then with *bar* probes. The membrane was rehybridized with *Act1* probe used as internal standard. (**D**) Analysis of the relative expression of both transgenes using semi-quantitative RT-PCR. The expression of the native rice *Act1* gene was used as internal standard. (**E**) Restriction digestion analysis of cDNA-amplified PCR products showing the difference between the expression of native and trans-*AHAS* genes and simultaneous confirmation of the introduced P171S mutation. The digestion of PCR products from transgenic plants yielded three bands of 401, 294 and 107 bp molecular weight while no digestion occurred in the *wt* plants. *wt*: wild type, L: Line number of transgenic plants (Full images of all the blots and gels are available in supplementary information from Figures SI5–SI8).

The *in silico* analysis revealed that the amino acid at position 171 in the rice AHAS protein is the conserved mutation point for herbicide tolerance; this amino acid was subsequently replaced by serine to develop herbicide-tolerant plants (Fig. 1A). While creating the P171S mutation in rice *AHAS*, the *Xba*I restriction site was introduced to help to distinguish transgenic plants from non-transgenic plant (Fig. 2E). We used the *Agrobacterium*-mediated transformation method for the generation of transgenic plants. The putative transgenic plants were initially screened for confirmation of the presence of transgene construct with the help of *bar* primers by PCR (Fig. SI1A and B). Since *AHAS* is a native gene, it was not possible to screen putative transgenic plants with *AHAS* primers because there would be no difference between native and transgenic PCR bands.

The PCR positive T1 transgenic plants were analyzed to confirm the presence of the transgene integration by southern blotting. The hybridization of nylon membrane with *AHAS* DIG labelled probe resulted in a common band for all the transgenic lines (including wild type (*wt*) control plant) (Fig. 2A); this band represents the native *AHAS* gene. In addition to native copy, there was an additional band found for all 5 transgenic lines (line 1, 2, 4, 5, 6), which showed the single copy *AHAS* transgene integration. Subsequently, the same nylon membrane was re-hybridized with *bar* DIG probe. The *bar* probe resulted in the same pattern as observed in *AHAS* hybridization except the common *AHAS* band due to the heterologous origin of *bar* gene (Fig. 2B). Among the transgenic plants (lines 1, 2, 4, 5 and 6), lines 4 and 6 showed similar band patterns for both the *AHAS* and *bar* genes; thus considered as the same transgenic events. Further, transgenic line 3 and the *wt* control did not show signals for any of the genes; this line was considered non-transgenic. Thus, the southern blot analysis confirmed a total of 4 different positive *AHAS* and *bar* single copy integrated independent transgenic events.

The expression levels of the *AHAS* and *bar* transgenes in all 4 southern-positive transgenic lines were further validated by northern blotting and semi-quantitative RT-PCR. Northern hybridization was performed consecutively with *AHAS* and *bar* DIG probes. Initially, the membrane was hybridized with *AHAS* DIG probe which resulted in an ~2 kb band for all the plant samples; however, the hybridization signal was found to be higher in all the transgenic lines compared to the *wt* controls (Fig. 2C). Furthermore, the membrane was reprobed with *bar* DIG probe which resulted in an ~500 bp hybridization signal for all transgenic lines; this band was absent in the *wt* control. The experiment confirmed the expression of both transgenes. Further, the expression of the *Act*I constitutive gene was used as an internal standard which showed equal expression in all plant samples (Fig. 2C). Similarly, the semi-quantitative RT-PCR experiment showed a higher expression (almost 2-fold) of the *AHAS* gene in the transgenic lines compared to the *wt* control plants due to the active transcription of the *AHAS* transgene copy (Fig. 2D). All four transgenic lines showed expression of the *bar* gene in semi quantitative RT-PCR which was absent in *wt* control plants. The expression of the *Act*I gene was used as the internal standard to confirm equal quantities of cDNA used in all the experiments which showed its uniform expression in all plant samples (Fig. 2D).

The restriction digestion analysis of semi quantitative RT-PCR product of the *AHAS* gene from all the transgenic and *wt* control plants further helped to distinguish between the expression of *OsmAHAS* and *OsAHAS*. The internal *AHAS* gene primers were designed to amplify a region flanking the P171S (*Xba*I) mutation site. A 401 bp PCR band was amplified from all the transgenic and *wt* plants using cDNA and was digested with *Xba*I restriction enzyme. The PCR product of *wt* plant could not digested with *Xba*I restriction enzyme while all the other transgenic lines showed three distinct bands with 107, 294 and 401 bp DNA products. The PCR-based strategy was used as a molecular marker to estimate the relative expression of the native *AHAS* versus trans-*AHAS* genes in all transgenic lines (Fig. 2E).

### Transgenic plants showed an increased tolerance to BM

**Figure 3:**
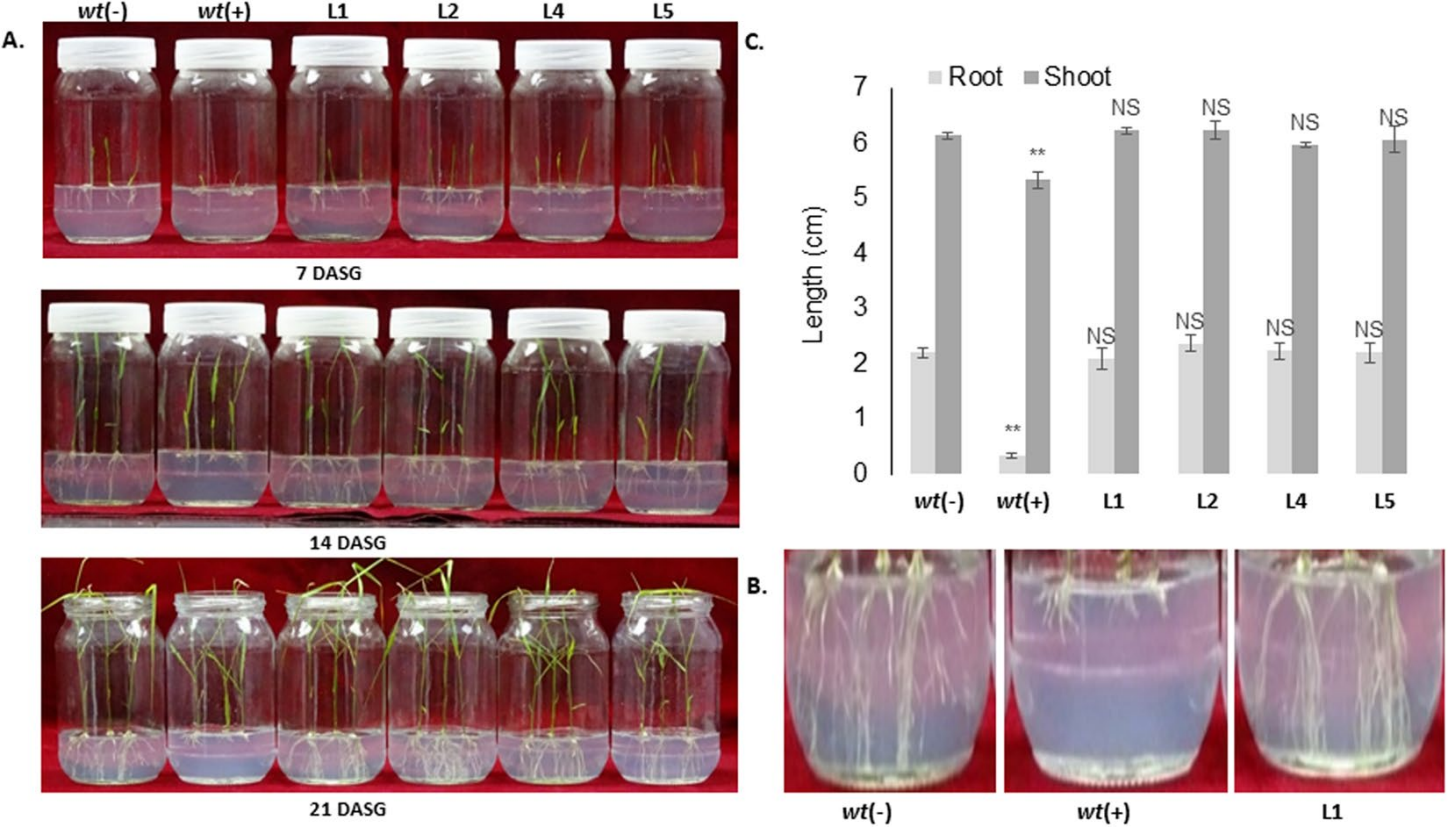
Physiological analysis of the tolerance of transgenic plants to BM herbicide at the seed germination stage. (**A**) Transgenic lines (L1, L2, L4 and L5) grew normally in the 100 μM BM supplemented media with root and shoot growth similar to those of *wt* (−) plants. However, the root growth of *wt* (+) plants was severely arrested at this concentration, while no shoot length was affected, which was comparable to *wt* (−) plants. (**B**) Comparison of the effects of BM on root growth in *wt* and transgenic line 1. (**C**) Graphical representation of the root-shoot length ratio. *wt* (−): wild type without herbicide treatment, *wt* (+): wild type treated with herbicide, L: Line number of transgenic plants, DASG: Days after seed germination.

**Figure 4:**
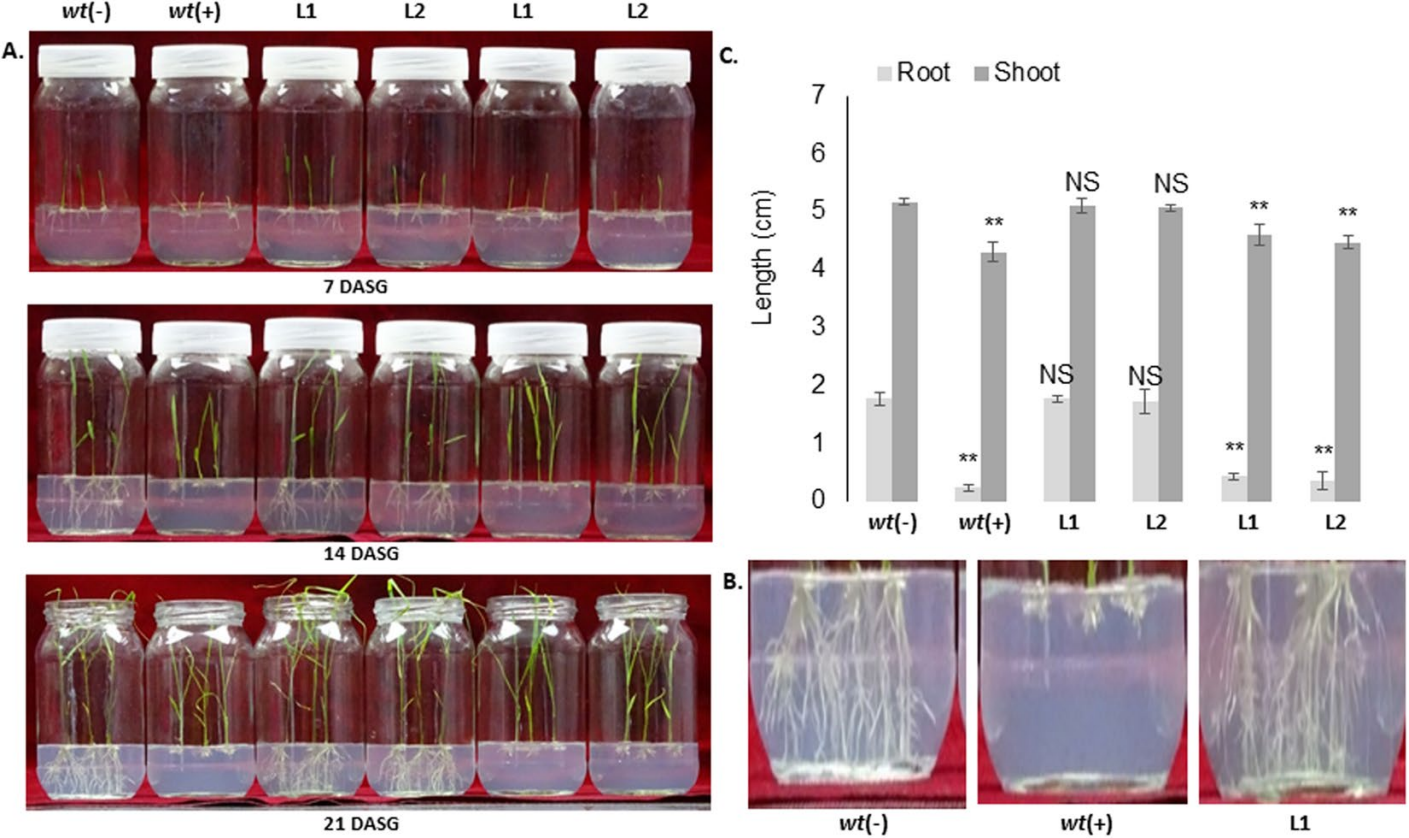
Comparative analysis of herbicide tolerance of transgenic plants to BM and BS at seed germination stage. (**A**) Effects of 100 μM BM and BS herbicides on seed germination and plant growth. Transgenic lines 1 and 2 efficiently tolerated to BM herbicide and showed normal phenotype similar to *wt* (−) plants. On the other hand, the root growth of transgenic lines 1 and 2 were significantly inhibited in BS supplemented media similar to *wt* (+) plants. (**B**) Comparison of root lengths between *wt* and transgenic line 1. **(C)** Graphical representation of the root-shoot length ratios of *wt* and transgenic lines. *Wt* (−): wild type without herbicide treatment, *wt* (+): wild type treated with herbicide, L: Line number of transgenic plants.

The tolerance mutations of AHAS exhibit different degree of resistance to various classes of AHAS-inhibiting herbicides. Among all the tolerance mutations, the amino acid substitution at position P171 in the AHAS protein is frequently observed in many tolerant weed biotypes imparting a higher degree of tolerance to sulfonylureas (SU) and relatively lower level of tolerance to pyrimidinylcarboxylates (PC) herbicides^22^. To validate the level of tolerance offered by the introduced P171S mutation in rice *AHAS*, the transgenic rice seeds were screened against two popular AHAS-inhibiting herbicides BM (belongs to SU) and bispyribac sodium (BS; belongs to PC) at the germination stage. Initially we checked the natural tolerance of control rice seeds against various concentrations of BM and BS. The results showed a complete inhibition of root growth at 25 μM concentration for BM and 20 μM for BS. There was no significant difference in shoot growth observed (data not shown). The surface sterilized T3 homozygous seeds were inoculated on half-strength MS media containing 100 μM concentration of BS or BM herbicides (Figs 3 and 4). The *wt* seeds treated with and without herbicides were used as positive (+) and negative (−) controls respectively, and handled alike. In BM supplemented growth media, all the transgenic lines showed vigorous root growth which was comparable to that of the *wt* (−) control, as recorded after 7, 14 and 21 days of seed germination (Fig. 3). Further, the *wt* (+) control showed poor root growth even after 14 days; however, no significant difference in shoot lengths was observed which was similar to the *wt* (−) control. The root growth of the *wt* (+) control plants was completely inhibited after the 7^th^ day, and no subsequent growth was observed (Fig. 3A, 3B).

To compare the degree of tolerance offered by the transgenic lines to sulfonylureas (SU) and pyrimidinylcarboxylates (PC) class of herbicides, the T3 transgenic lines (1 and 2) were grown in half strength MS media supplemented with 100 μM BM and BS separately. The root growth of these transgenic lines in the BM supplemented media showed normal morphology, which was comparable to that of *wt* (−) controls (Fig. 4). However, the root growth was significantly arrested in both the transgenic lines in the BS media which was comparable to that of *wt* (+) plants (supplemented with 100 μM BS). Although, the transgenic plants showed a moderate level of tolerance to 50 μM BS at germination stage (data not shown). Furthermore, we did not observe any significant shoot growth differences among the transgenic lines, including the *wt* (−) and *wt* (+) controls, even at 21 days after seed germination (Fig. 4A–C).

The tolerant transgenic lines (1 and 2), which were confirmed by seed germination tests, were further examined to determine the effect of BM herbicide on the overexpressed mutant *OsmAHAS* gene in transgenic plants via color tests. Transgenic lines 1 and 2 developed a light red color due to the accumulation of acetoin resulting from the enzymatic action of mutant OsmAHAS proteins in the presence of 0.1 µM BM. The color of the *wt* (−) control reaction was dark red because of the uninhibited accumulation of high acetoin due to the lack of BM. However, the color of the *wt* (+) control reaction remained yellowish due to the presence of the inhibitor BM and the absence of mutant OsmAHAS. The native OsAHAS protein is unable to produce acetolactate due to the inhibitory action of BM, thus leading to no acetoin formation and the color of the reaction remained yellow (Fig. SI2A). The results showed active expression of the mutant *OsmAHAS* gene.

### Confirmation of transgenic lines tolerance to phosphinothricin herbicide

**Figure 5:**
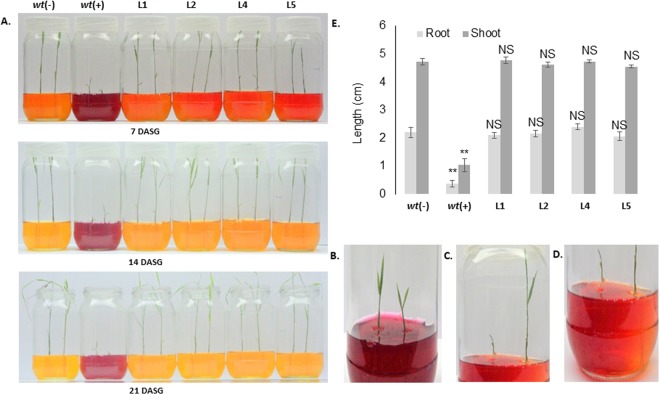
Effects of phosphinothricin on transgenic plants at the seed germination stage. (**A**) Effect of 30 mg/L of phosphinothricin herbicide on the growth of transgenic and *wt* plants. All the transgenic plants survived in phosphinothricin and changed the color of the media from deep red to yellow; however, the *wt* (+) plants could not grow much and the color of media remained the same. (**B**) Stunted growth of wild-type plants in the presence of phosphinothricin. (**C**) Effects of phosphinothricin on the shoot growth of *wt* and transgenic plants. (**D**) Figure showing variation in the media color by *wt* and transgenic line. (**E**) Graphical representation of root-shoot length ratios of transgenic and *wt* plants in the presence of phosphinothricin herbicide.

Chlorophenol red is a color indicator dye that appears red at pH 6 and turns yellow with a decrease in pH. To validate the activity of the *bar* gene in transgenic plants, seeds from the *wt* control and transgenic groups were germinated on half strength MS media supplemented with 30 mg/L phosphinothricin herbicide. We observed a gradual change in media color from red to yellow after 10 days of seed inoculation in all transgenic lines, indicating active expression and function of the *bar* gene. The bar protein detoxified phosphinothricin rapidly which resulted in healthy growth of all transgenic plants (Fig. 5A–D); this result was comparable to that of the *wt* (−) controls. On the other hand, the *wt* (+) control plants were unable to detoxify phosphinothricin due to the absence of the *bar*, and consequently the media color remained unchanged (Fig. 5A,B). Unlike BM and BS herbicide, phosphinothricin has equal effects on both root and shoot growth (Fig. 5E). The root and shoot development was severely arrested in *wt* (+) plants while the transgenic plants showed normal growth.

We tested the effective tolerance of transgenic plants against commercially available phosphinothricin (marketed as ‘Basta’) during the vegetative stage. Basta (in which phosphinothricin is the active ingredient) is a contact herbicide that has a localized toxic effect on plant tissue. Phosphinothricin irreversibly binds to glutamine synthetase (GS), an important enzyme in the ammonia detoxification mechanism. The transgenic lines as well as control plants were painted (on leaf tip region) with 3% (v/v) basta herbicide at the 8–12 leaf stage. The localized toxic effect of basta killed the control plant leaves within 10 days of application due to the accumulation of ammonia, however, the transgenic lines (except line 4) did not show any leaf necrosis due to the detoxification of phosphinothricin by bar (Fig. SI2B). Phosphinothricin irreversibly binds to glutamine synthetase (GS), an important enzyme in the ammonia detoxification mechanism, and results in plant death.

### Characterization of transgenic plants under the foliar application of BM and basta herbicides

**Figure 6:**
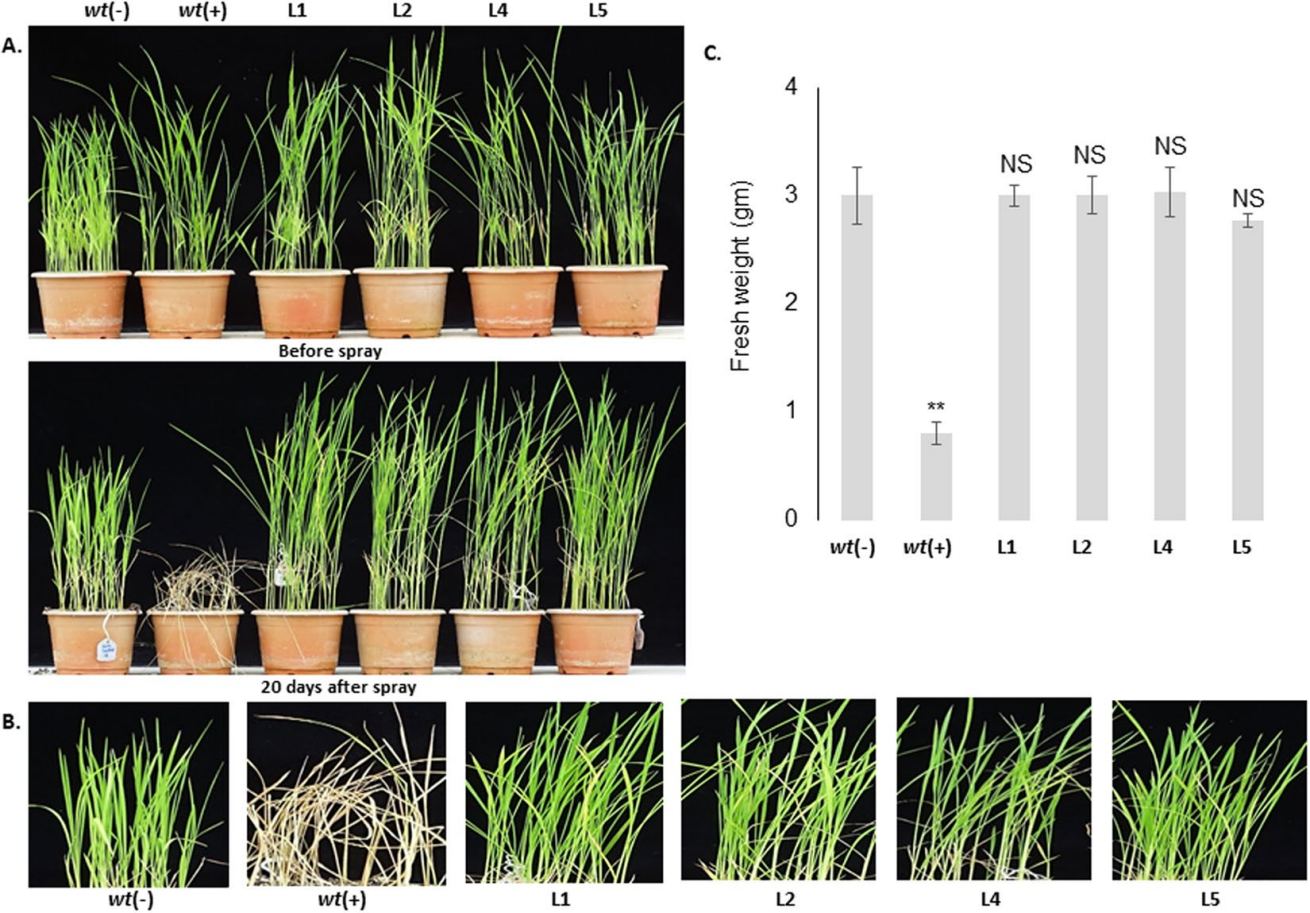
Effects of foliar spraying of BM herbicide on transgenic plants. (**A**) The transgenic plants efficiently tolerated 300 μM BM herbicide application without any visible necrotic symptoms while the *wt* (+) plants died shortly thereafter. (**B**) Effects of BM herbicide application on leaves. (**C**) Graphical representation of gram fresh weight of herbicide treated transgenic and *wt* plants.

The T3 homozygous transgenic lines as well as two *wt* control plants (one month old) were analyzed for their field level tolerance against the herbicides BM and basta. The first set of transgenic lines and *wt* controls were foliar sprayed with 300 μM BM (Fig. 6A, 6B). Necrotic symptoms started on *wt* (+) control plants after 4–5 days of BM application, and the leaves turned yellow and completely died after 15–20 days. However, all four transgenic lines showed healthy growth without any necrotic symptoms in response to the same BM application; their leaves stayed green which was comparable to the *wt* (−) control plants (without BM application) (Fig. 6A, 6B). There was no significant difference between the transgenic and *wt* (−) plants in terms of growth and morphology (Fig. 6C). The measurements of the total fresh weight of the herbicide treated transgenic lines did not show any growth penalty which suggests that the introduced P171S mutation added an advantage to the transgenic plants in providing tolerance to BM herbicide.

**Figure 7:**
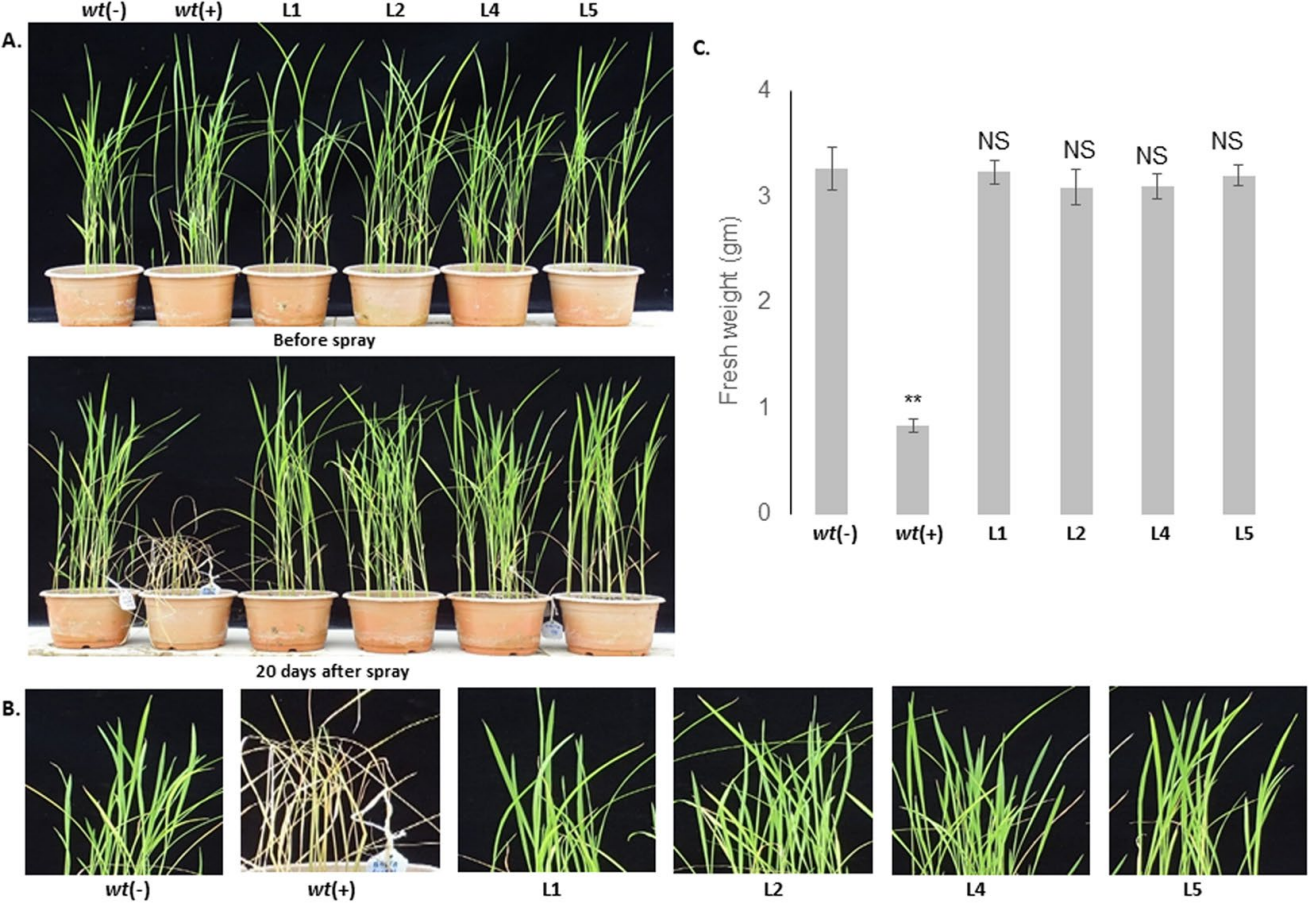
Effects of foliar application of basta herbicide on transgenic plants. (**A**) Transgenic plants showed significant tolerance to 2% basta and were healthy even after 20 days of application, while the *wt* (+) plants died shortly thereafter. (**B**) Effects of basta on the leaf of *wt* and transgenic lines. (**C**) Graphical representation of total biomass of basta treated plants at 20 days after application. The graph shows no significant difference between the transgenic and *wt* (−) plants in terms of phenological attributes.

Similarly, a second set of plants (transgenic lines and *wt* control) were sprayed with 2% (v/v) commercially available basta solution. The transgenic lines showed normal morphology with healthy leaves without any leaf necrosis similar to *wt* (−) plants. Furthermore, the total fresh biomass was analyzed which did not show any noticeable differences between the transgenic and *wt* (−) control plants, indicating no morphological penalty on the transgenic plants due to the active expression of *bar* (Fig. 7C). However, the *wt* (+) plants started showing necrotic symptoms after 6–7 days and completely died within 10–15 days (Fig. 7A, 7B).

**Figure 8:**
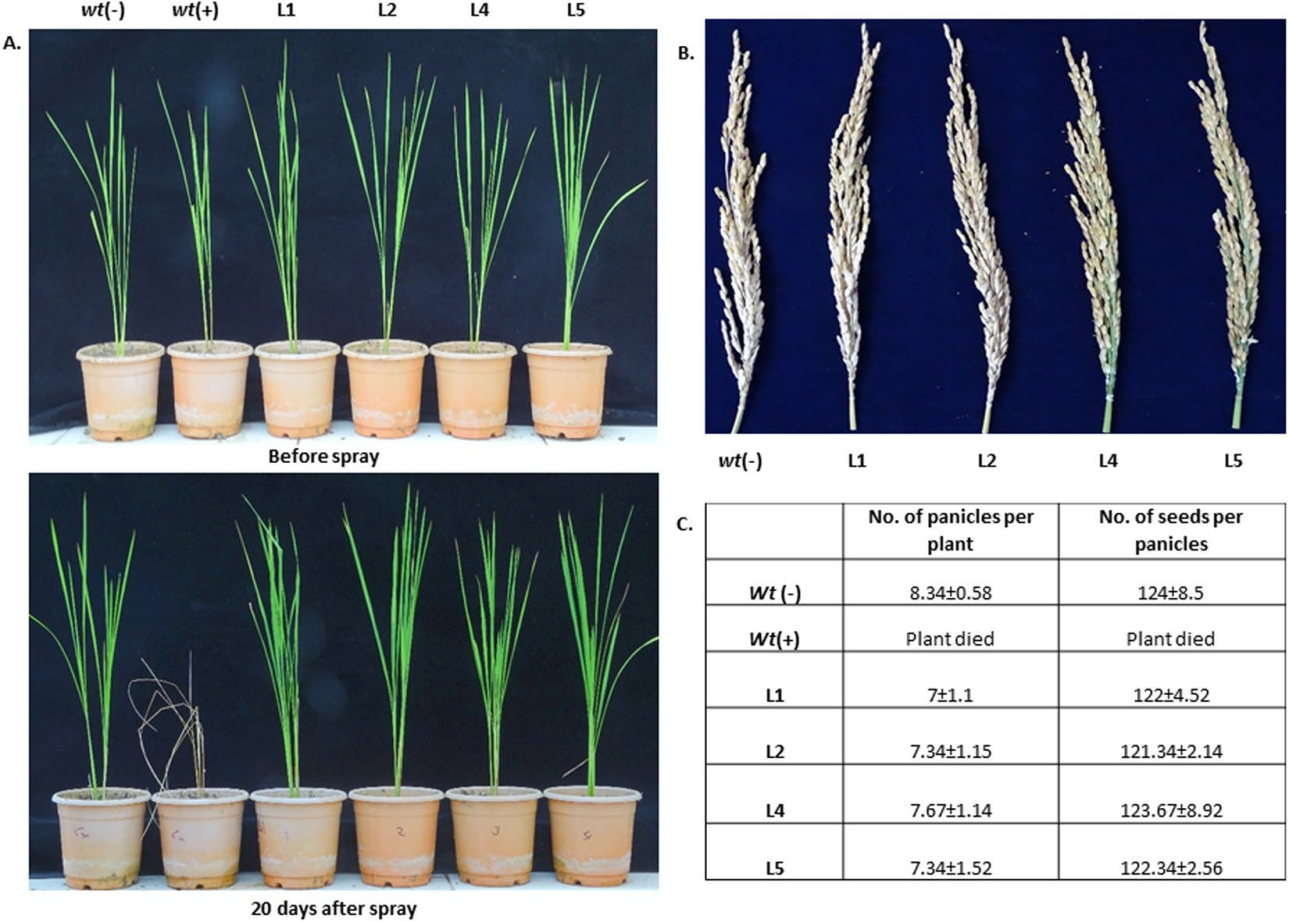
Analyzing the dual herbicide tolerance of transgenic plants. (**A**) Transgenic plants were sprayed with 300 μM BM after 7 days of application of 2% basta. No phenological symptoms of leaf injury was recorded in transgenic lines, while the *wt* (+) plants died shortly even before the application of BM. (**B**) Effects of transgene integration and dual herbicide applications on the yield of transgenic lines. (**C**) Yield analysis of number of the seeds per panicle of transgenic (after herbicide application) and *wt* (−) plants. No significant differences were observed between the *wt* (+) and transgenic plants.

We also examined the effects of dual applications of the herbicides basta and BM on the transgenic plants. A set of transgenic lines and *wt* control plants were initially sprayed with a 2% (v/v) basta solution. At 7 days of basta application, the same set of plants was similarly sprayed with 300 μM BM. There was no phenological injury observed in the transgenic lines which remained green and healthy even at 20 days post-application (Fig. 8A). Further, we allowed the transgenic plants to grown until the maturity stage to determine the effects of dual applications of herbicides on yield. The transgenic plants did not show any yield penalty having normal seed setting and seed number per panicle which was similar to the *wt* (−) control plants (Fig. 8B, 8C).

## Discussion

To overcome the problem of frequent evolution of herbicide-resistant weeds, the alternating or combined use of multiple herbicides in conjunction with traditional practices is always suggested. The alternating or combining of herbicides provides the least chance for a weed species to acquire resistance against respective herbicides in such a short span of time^23^. Thus, developing transgenic plants that have more than one herbicide tolerance could be a great solution to overcome this problem. However, only a few genetically modified crop plants that are tolerant to multiple herbicides have been reported in the public and private sectors. There is only a single report from the public sector, to the best of our knowledge, mentioning the development of PGMS (photoperiod-sensitive genic male-sterile) transgenic rice which was engineered with dual herbicide tolerance traits against the glyphosate and glufosinate herbicides^24^. A double herbicide tolerant soybean event 356043 that contained a mutated soybean *gm-hra* (*Glycine max herbicide*-*resistant acetolactate synthase*) and glyphosate resistance gene was developed by Pioneer Hi-Bred, Johnston, IA which was deregulated in 2008 by the United States Department of Agriculture (USDA). Subsequently, the company has also planned to make transgenic corn with the same herbicide traits. The double (HPPD and glyphosate) and triple (HPPDi, glyphosate and glufosinate) herbicide tolerant transgenic soybean have been claimed to be developed or it is in pipeline by Bayer Crop Science. With the large demand of soybean in the global market, another global competitor Monsanto (St. Louis, MO) has developed transgenic soybean events against glyphosate and dicamba (auxinic herbicide) along with a 3^rd^ mode of action, these are under field trials and referred as 3^rd^ Generation HT:RR Xtend^TM^ Crop System. Dow Agro Sciences (Indianapolis, IN) has also planned to commercialize transgenic corn, cotton and soybean that exhibit tolerance to glyphosate along with 2, 4-dichlorophenoxyaceticacid (2, 4-D) (Enlist^TM^), with both 2,4-D and glufosinate (Enlist E3^TM^) and together with aryloxyphenoxypropionates herbicides. In addition, Syngenta and Bayer Crop Science have in their pipeline to develop dual herbicide tolerant soybean against HPPDi and glufosinate^4,11,25,26^.

Here, we reported the development of dual herbicide tolerant transgenic indica rice (cultivar Swarna) against BM and basta herbicides for sustainable weed management. There have been 22 amino acid substitutions at seven different sites with in the AHAS reported in many tolerant weeds. The common mutation point P197 (equivalent to P171 in rice) is quite vulnerable to 11 different amino acid substitution mutations that provide resistance against several AHAS-inhibiting herbicide compounds and occurs very frequently in AHAS-inhibiting herbicide resistant biotypes^1^. Previously, transgenic plants generated by overexpressing AHAS with P197S mutation exhibited a high degree of tolerance against herbicide bensulfuron methyl (BM) and moderate resistance against bispyribac sodium (BS)^27^. Similar to the above finding, in present study, the transgenic lines showed tolerance to 100 µM BM and exhibited normal phenotype; while a complete root growth inhibition was observed in these plants in 100 µM BS supplemented media (Figs 3 and 4). Odell and colleagues reported that the mutated form of *AtAHAS* overexpressed in transgenic plants showed a 300-fold increased tolerance against chlorsulfuron while the wild type *AtAHAS* gene conferred only 3-fold tolerance to those plants^28^. Similarly, Kawai and coworkers compared nine different kinds of P171 rice AHAS mutations for their sensitivities against sulfonylurea herbicides. The authors confirmed the high resistance of the P171S mutation against BM herbicide with an RS ratio greater than 8000^29^. Yu and colleagues conducted a similar study in which they compared and functionally validated the pleotropic effects of four different P197 substitution mutations of AHAS in *Lolium rigidum*. Among the mutations, P197S showed no effects on AHAS kinetics and subsequently had no penalty on overall plant growth. The study further highlighted the preference for a serine substitution for proline in AHAS over any other mutation in nature^22^. Mutant variants of AHAS against BM and related herbicide derivatives have been well documented in many crops such as rice^27,30–33^, soybean^34^, sugarcane^35^ and wheat^36^. In 1992, Sengnil and coworkers isolated BM-tolerant rice cells from cell suspension culture by continuously growing rice cells in herbicide medium. These cells were tolerant to 10^−6^–10^−5^ M concentration of BM^37^.

Basta is a contact herbicide that irreversibly inhibits the glutamine synthetase (GS) enzyme resulting in the accumulation of ammonia in the cell and subsequent plant death. The bacterial *bar* gene is responsible for the detoxification of phosphinothricin herbicide by acetylating its amino group and helps in the survival of the plants^38–41^. This gene has been commonly used in many herbicide-tolerant transgenic crops, including rice^24^, tobacco^20^, soybean^42^, maize^43^ and cotton^44^.

The AHAS and bar enzymes have different mechanisms of action against their respective herbicides; these mechanisms are very effective. We developed four independent dual herbicide-tolerant transgenic indica rice (Swarna) lines against BM and phosphinothricin (basta) herbicides. The molecular analysis confirmed the expression of both transgenes in all transgenic plant lines. The seed germination analysis of *wt* control rice plants revealed its natural tolerance to BM up to 25 µM and tolerance to BS up to 20 µM concentrations which is relatively high compared to other indica and japonica rice varieties. Previously, two indica, five indica-derived and one japonica rice variety were checked for their tolerance limit against BS herbicide^30^. With tolerance limit up to 2.5 µM BS concentration (moderately inhibited) at the seedling stage, the *wt* indica rice cultivar Kasalath was the most tolerant among the selected cultivars, although its roots were completely inhibited at 0.5 µM. However, all the other cultivars showed high susceptibility to 2.5 µM concentration. There are no previous reports regarding the effects of BM herbicide on the seed germination of rice to the best of our knowledge. AHAS color test on crude enzyme extracts showed functional activity of OsmAHAS trans-protein in the transgenic lines in the presence of 0.1 µM BM. Further, the transgenic lines were able to tolerate 300 µM BM spraying at the 8–12 leaf stage. Shimizu and colleagues transformed tobacco plants with a mutated *Arabidopsis* P197S *AHAS* gene and generated transplastomic plants. These plants were able to show functional activity of trans-AHAS proteins in the presence of 0.1 µM BM herbicide in color tests, and the excised tobacco leaves were also able to survive on MS media that were supplemented with the same concentration of BM herbicide^45^. A mutant P169S *Monochoria vaginalis AHAS* gene was used by Song and colleagues to transform *Arabidopsis*. The transgenic plants were able to tolerate a high concentration of BM herbicide, as confirmed by hypocotyl length estimations and color test^46^.

Our transgenic lines were also able to detoxify 30 mg/L of phosphinothricin herbicide as recorded at the seed germination stage. In addition, the generated transgenic plants showed high tolerance to 2% and 3% basta herbicide as confirmed by foliar spraying and leaf paint assay respectively. The application of basta to whole plants at the vegetative stage showed no visible injury and fitness costs to the transgenic plants. Similar results have been reported in previous studies involving the *bar* in different crop plants^20,42,47,48^. This investigation highlights the use of effective simultaneous dual herbicide tolerance of transgenic rice plants without causing any yield penalty. However, additional experiments are needed to analyze the tolerance against both herbicides under natural field conditions.

## Conclusion

We have successfully developed dual herbicide-tolerant transgenic rice plants which were confirmed at various plant developmental stages i.e. at the seed germination, mature leaves and seedling stages; the transgenic plants showed a higher level of tolerance against BM and basta herbicides. Further, the transgenic plants performed well without any yield penalty. This study highlights the importance of the P171S mutation for imparting herbicide tolerance and the development of dual herbicide-tolerant transgenic plants which will add additional knowledge to weed management practices of rice cultivation.

## Methods

### *In silico* analysis of AHAS-inhibiting herbicide tolerant P to S mutation in rice

A total 16 of AHAS protein sequences from various plant species i.e. *Oryza sativa*, *Alopecurus myosuroides*, *Amaranthus hybridus*, *Amaranthus palmeri*, *Amaranthus viridis*, *Amaranthus rudis*, *Apera spica-venti*, *Arabidopsis thaliana*, *Bacopa rotundifolia*, *Chenopodium album*, *Conyza canadensis*, *Glycine max*, *Kochia scoparia*, *Lolium multiflorum*, *Solanum lycopersicum* and *Zea mays*, were aligned to determine the position of the tolerant P197S substitution in rice with the help of MEGA 6.0 tool.

### Introduction of P171S tolerant mutation in rice *AHAS*

The mutation P171S in the rice *AHAS* gene was introduced via PCR based site directed mutagenesis (refer to supplementary information for details).

### Preparation of gene construct and generation of transgenic plants

The final gene construct with mutated *AHAS* and *bar* genes was cloned into the pMDC99 Gateway compatible cloning vector and transformed into *Agrobacterium* strain EHA 105, which was further used for transformation in rice calli (refer to supplementary information for details) (Fig. 1B, 1C).

### Molecular and expression analyses of putative transgenic plants

The tissue culture generated putative transgenic plants were confirmed by PCR and southern blotting, and the expression analysis of the confirmed transgenic plants was performed by northern blotting and semi-quantitative RT-PCR. The presence and expression of the mutaned *AHAS* gene was also confirmed by restriction digestion analysis (refer to supplementary information for details).

### Analysis of herbicide tolerance during the seed germination stage

To verify the level of tolerance offered by the overexpression of *OsmAHAS*, the natural tolerance of control Swarna plants against BS and BM herbicides were analyzed by growing the sterilized seeds on half-strength MS media that contained the various concentrations of herbicides (BS and BM). The concentrations of herbicides used were 0, 10, 20, 30, 40, 50 and 100 μM. The seeds were surface sterilized and grown for 15–20 days to regularly monitor the effects of the herbicides on plant growth. The tolerance of the transgenic plants at the seed germination stage against these herbicides was analyzed by growing the T3 seeds in half-strength MS media supplemented with 100 μM BS or BM herbicides separately. In both experiments, the positive and negative *wt* controls were also maintained along with the transgenic lines.

To assess the activity of the *bar* at the seed germination stage, the sterilized seeds of all four transgenic lines, in addition to the positive and negative *wt* control lines, were inoculated on half-strength MS media (pH 6.0) with 50 mg/L of chlorophenol red (CR) and 3 mg/L phosphinothricin herbicide. The *wt* seeds inoculated on the media with phosphinothricin served as positive controls, while the *wt* seeds germinated on media without phosphinothricin were used as negative controls.

### Characterization of transgenic plants during the vegetative stage

The toxic level of BM herbicide was initially assessed by foliar application of various concentrations of BM including 50, 100, 150, 200, 250 and 300 μM on one month-old *wt* control plants, and the lethal dose was chosen based on the appearance of necrotic symptoms on the leaves. Two separate sets of all four T3 homozygous transgenic lines along with *wt* control plants were grown to analyze the field level tolerance against both herbicides i.e. BM and basta. The plant groups were foliar sprayed with respective herbicides and the results were monitored based on the appearance of injury symptoms.

The first set of transgenic plants were treated with 300 μM BM (tissue culture grade) solution using 0.1% Tween-20 as a surfactant and the second set with 2% (v/v) basta herbicide (Bayer Crop Science, Germany). The third set of transgenic plants were sprayed with both herbicides BM and basta at an interval of 7 days. The negative control *wt* (*wt* (−)) plants in all experiments were sprayed with water along with 0.1% Tween-20 while the positive *wt* (*wt* (+)) plants were sprayed with the same concentrations of the respective herbicides. The results were monitored regularly at 7-days intervals in each experiment.

For these experiments, a total of 20 plants were taken from a particular group and subjected to herbicide treatment. The herbicide-treated plants were analyzed and compared for any phenological injury or impact of transgene integration on the grain yield between transgenic plants and *wt* (−) plants.

### Leaf paint assays and AHAS color tests

The leaf paint assay and AHAS color tests were performed on mature leaves of transgenic plants to confirm the activity of bar and AHAS proteins respectively (refer to supplementary information of full detail).

## Electronic supplementary material

Supplementary Information
