# Supplementary material for: Developing dual herbicide tolerant transgenic rice plants for sustainable weed management
Source: Sci Rep. 2018 Aug 2;8:11598. doi: 10.1038/s41598-018-29554-9 (PMC6072789; doi:10.1038/s41598-018-29554-9)
Supplement: Supplementary file 1 — Supplementary Information [file 41598_2018_29554_MOESM1_ESM.docx]

**Developing dual herbicide tolerant transgenic rice plants for sustainable weed management**

Dhirendra Fartyal^1,2^, Aakrati Agarwal^1,3^, Donald James^1^, Bhabesh Borphukan^1^, Baburam^1,2^, Vijay Sheri^1^, Pawan K. Agrawal^4^, V. Mohan Murali Achary^1,*^ and M. K. Reddy^1,*^

^1^Crop Improvement group, International Centre for Genetic Engineering and Biotechnology, Aruna Asaf Ali Marg, New Delhi, India, 110067.

^2^Uttarakhand Technical University, Dehradun, Uttarakhand, India

^3^Plant Molecular Biology Lab, Department of Botany, University of Delhi, New Delhi, India

^4^National Agricultural Science Fund, Indian Council of Agricultural Research, New Delhi, India

* **Correspondence**

Malireddy K. Reddy

[reddy@icgeb.res.in](mailto:reddy@icgeb.res.in)

V. Mohan Murali Achary

[achary.mohan1@gmail.com](mailto:achary.mohan1@gmail.com)

**Table SI1. List of primers used in the study.**

| **S. No.** | **Primer Name** | **Sequence** |
| --- | --- | --- |
| 1. | AHAS-AF | 5’- CCCCTTAATGATAGGAGGGCTC-3' |
| 2. | AHAS-AR | 5’- CATGCGTCTAGAGACCTGGCCCGTGATGGC -3’ |
| 3. | AHAS-BF | 5’- GCCATCACGGGCCAGGTCTCTAGACGCATG -3’ |
| 4. | AHAS-BR | 5’- GCATAGAAGTACTTTATTCTCTT-3’ |
| 5. | CaMV-F | 5’ TGAGACTTTTCAACAAAGGGTA 3’ |
| 6. | CaMV-R | 5’ GATCTGGATTT TAGTACTGGAT 3’ |
| 7. | Bar-F | 5’ATGAGCCCAGAACGACGCCCGGCCGA 3’ |
| 8. | Bar-R | 5’-TCATCAGATCTCGGTGACGGGCAGGA-3’ |
| 9. | AHAS-F | 5’ GCGCGGACATCCTCGTGGAG 3’ |
| 10. | AHAS-R | 5’ GGGGATGTCCTCCACATCAA 3’ |

**METHODS**

**Introduction of P171S tolerant mutation in rice *AHAS***

*In silico* analysis revealed the universal hotspot and conserved position of amino acid proline at 171 in rice AHAS polypeptide which is , identified in many herbicide tolerant biotypes to be changed into serine. A full length 3229 bp of *AHAS* gene cassette was PCR amplified from indica rice cultivar Swarna containing 1 kb of *AHAS* native promoter sequence, 1935 bp of *AHAS* coding sequence and 294 bp *AHAS* 3’UTR in two parts. The method was designed to simultaneously amplify and introduce P171S mutation in *AHAS* gene with the help of PCR directed mutagenesis. A 1522 bp of DNA fragment was amplified by using primers AHAS-AF and AHAS-AR containing 1 kb region of promoter and 522 bp region of *AHAS* gene. Similarly, the second part of expression cassette of1737 bp was amplified by using primers AHAS-BF and AHAS-BR flanking 1425 bp of remaining portion of *AHAS* gene along with 294 bp of 3’UTR (refer to supplementary Table SI1 for all primers detail). Both the PCR amplified fragments were purified, restriction digested with *Xba*I, ligated and finally cloned into Gateway^®^ compatible entry vector 1 (EV1).

**Preparation of gene construct and generation of transgenic plants**

The *bar* gene expression cassette (*BAR*-EV2), under the regulation of CaMV 35S promoter and its 3’UTR, was amplified from the vector pMDC123 with the help of primers CaMV-F and CaMV-R and cloned in *Eco*RI and *Hind*III polylinker sites of entry vector 2 (EV2). To make double herbicide tolerant gene construct, the *OsmAHAS*-EV1 expression cassette was initially cloned into pMDC99 plant transformation vector, and the resulting pMDC99-*OsmAHAS* plasmid was subsequently used for cloning of *BAR*-EV2 expression cassette following Gateway LR cloning protocol (Fig. 1B and 1C). The pMDC99-*OsmAHAS*-*BAR* plant transformation vector was subsequently transformed into *Agrobacterium* EHA-105 strain by electroporation method, and PCR confirmed clones were used for rice transformation (Fig. 1C).

The recombinant pMDC99-*OsmAHAS*-*BAR* gene construct was, further, transformed into healthy, embryogenic and young calli of rice via *Agrobacterium* mediated transformation (Fig. SI1A). After three rounds of hygromycin (50 mg/L) selection, the resulted secondary calli were used for regeneration of young plantlets. After proper rooting and sufficient growth, the putative transgenic plants were transferred to green house.

**Molecular analysis of putative transgenic plants**

Since, it was difficult to screen the *OsmAHAS* transgene due to the presence of native *AHAS* copy in the rice genome, all the putative transgenic plants were screened for the presence of 532 bp *bar* transgene. The full-length primers Bar-F and Bar-R were used for transgenic screening with the help of PCR using the cycle 94^0^C for 1 min, 57^0^C for 1 min and 72^0^C for 1 min with an initial denaturation of 4 min at 94^0^C and a final extension of 10 min at 72^0^C.

The southern blot analysis of putative transgenic plants was performed to identify the unique transgene integration events according to Manna and colleagues^1^. Briefly, the RNA free pure DNA was isolated from all transgenic and *wt* plants, and completely digested with restriction enzyme *Bcl*I. The digested product was size fractionated on 0.8 % agarose gel, denatured and subsequently transferred to nylon membrane. The membrane was initially hybridized with *AHAS* probe and consecutively reprobed with *bar* following manufacturer’s instructions to ascertain the presence of both genes.

**Expression analysis of transgenic plants**

The single transgene integrated transgenic lines, confirmed by southern blotting, were subjected to expression analysis by northern blotting and semi-quantitative RT-PCR. The northern blot analysis was carried out according to Chandrashekhar and coworkers^2^. In brief, a total RNA (15 µg) from transgenic and *wt* plants were used and run on denaturing 1% agarose gel containing formaldehyde. The agarose gel separated RNA fragments were subsequently transferred onto positively charged nylon membrane. The nylon membrane was first hybridized with *AHAS* probe, and subsequently reprobed with *bar* to confirm the transgene expression level as per manufacture’s instruction. The membrane was finally hybridized with *Act*1 gene probe which was used as internal reference gene.

The relative expression of transgenes was further confirmed with the help of semi-quantitative Real Time (RT) PCR. The specific gene primers were used to identify the relative expression of *AHAS* and *bar* in all the transgenic lines. The constitutively expressed *Act*1 gene was used as internal gene control. The template cDNA used in this experiment was made by reverse transcribing the total RNA (2 µg) from all transgenic and *wt* plants with the help of SuperScript III enzyme (Invitrogen). The PCR conditions used were as follows - 94^0^C for 1 min, 57^0^C for 1 min and 72^0^C for 1 min for 30 cycles, with an initial denaturation of 4 min at 94^0^C and a final extension of 10 min at 72^0^C.

**Expression analysis of mutant *AHAS* transgene**

The active expression of *OsmAHAS* in transgenic lines was confirmed by PCR amplification of 401 bp internal band along with region of P171S substitution using cDNA as template from all the transgenic and *wt* plants. The purified PCR products were digested with *Xba*I restriction enzyme and run on agarose gel to confirm the difference between the expression level of mutated *AHAS* transgene from the native *AHAS* gene.

**Leaf paint assay**

The herbicide resistance against basta was also analyzed by performing leaf paint assay. All the transgenic lines along with one control line were grown 8-12 leaf stage and the tip part of one leaf from each line was painted with 3 % (v/v) basta solution by a cotton swab on the upper part of the leaf. The leaf tip part of negative control line was also painted with only water on the leaf surface.

**AHAS color test**

Color test to see the activity of mutated *OsmAHAS* transgene was performed by following the method described by Gerwick and coworkers^3^ with some modifications. Briefly, the young leaves from all transgenic and *wt* plant lines were taken and macerated in liquid nitrogen in the presence of 50 mM phosphate extraction buffer. The plant material, thus obtained, was filtered and centrifuged at 15000 rpm for 20 mins, stored at 4^0^C which was further used as crude enzyme extract. For AHAS color assay, 500 μL of enzyme extract from wild type (for positive and negative) and transgenic plants (line 1 and 2) was added to their respective tubes. The positive control tube was then added with 500 μL of distilled water while negative control and transgenic tubes with 500 μL of 0.1 μM BM herbicide solution adding 250 μL 1.8 N H_2_SO_4_ only to negative tube. The reaction mixtures were thoroughly mixed and incubated at 37^0^C for 90 mins. The reactions were then stopped by adding 250 μL 1.8 N H_2_SO_4_ to all the tubes except negative one and incubated for 15 mins at 60^0^C. Next, 700 μL of 2N sodium hydroxide solution containing creatinine (0.25 %) and naphthol (2.5 %) was added to all the reaction mixtures to form colored complex and incubated for 15 mins at 60^0^C. The intensity of obtained colored complex was analyzed to assess the herbicide tolerance of transgenic lines.

**REFERENCES**

1. Manna, M., Achary, V. M. M., Islam, T., Agrawal, P. K. & Reddy, M. K. The development of a phosphite-mediated fertilization and weed control system for rice. *Sci. Rep.* **6,** 1–12 (2016). doi:10.1038/srep24941.
2. Chandrasekhar, K., Reddy, G. M. & Singh, J. Development of Transgenic Rice Harbouring Mutated Rice and Allium sativum Leaf Agglutinin ( ASAL ) Genes Conferring Tolerance to Herbicides and Sap-Sucking Insects. 1146–1157 (2014). doi:10.1007/s11105-014-0715-3.
3. Gerwick, B. C., Mireles, L. C. & Eilers, R. J. Rapid Diagnosis of ALS/AHAS-Resistant Weed. Weed Technol. 7, 519–524 (1993).

**FIGURES**


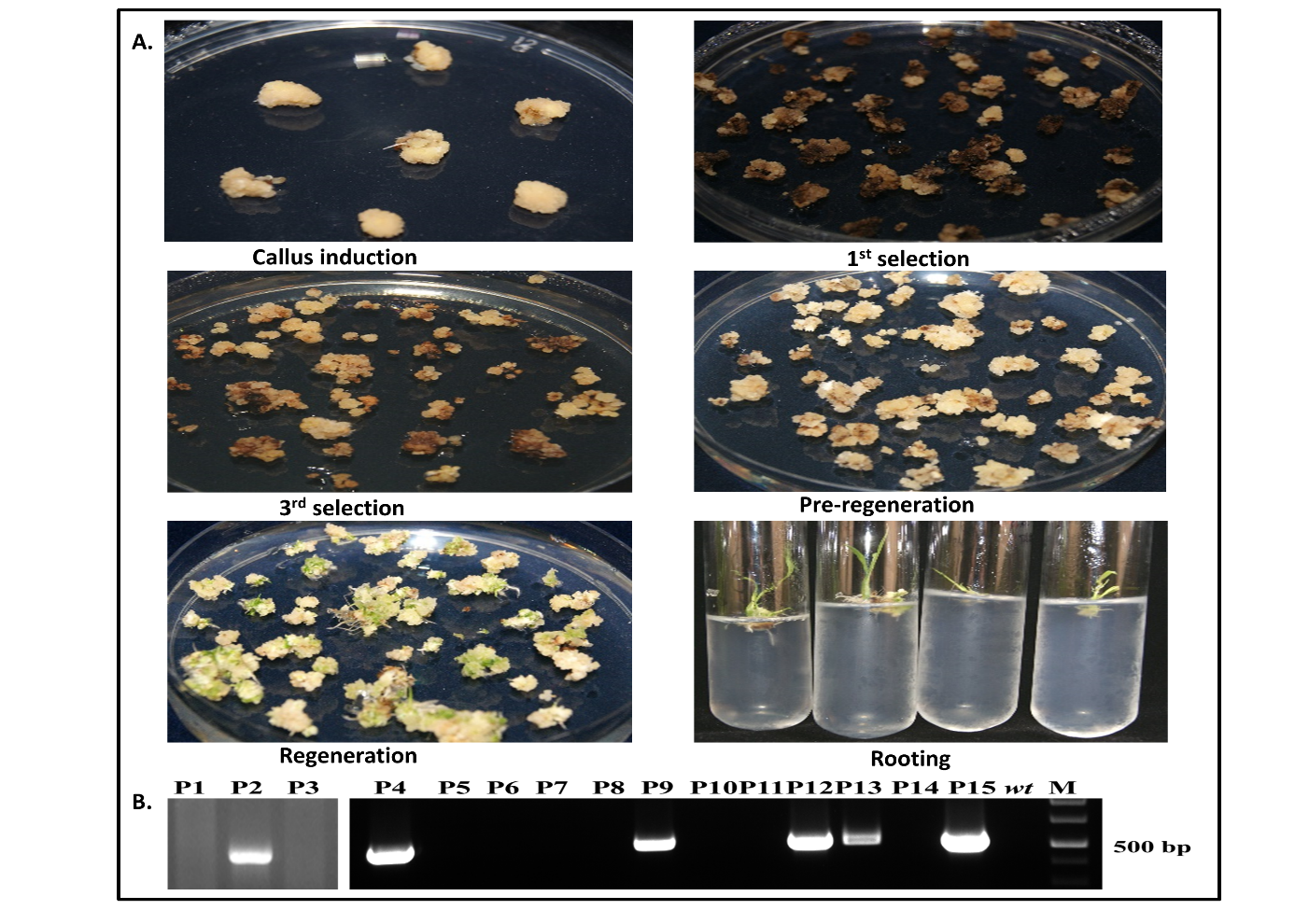


**FIGURE SI1. Generation of transgenic plants and their molecular confirmation. (A)** Various stages of tissue culture for generation of transgenic plants. **(B)** PCR confirmation of putative transgenic plants screened with *bar* primers. M: marker, *wt*: wild type plant, P: plant number.


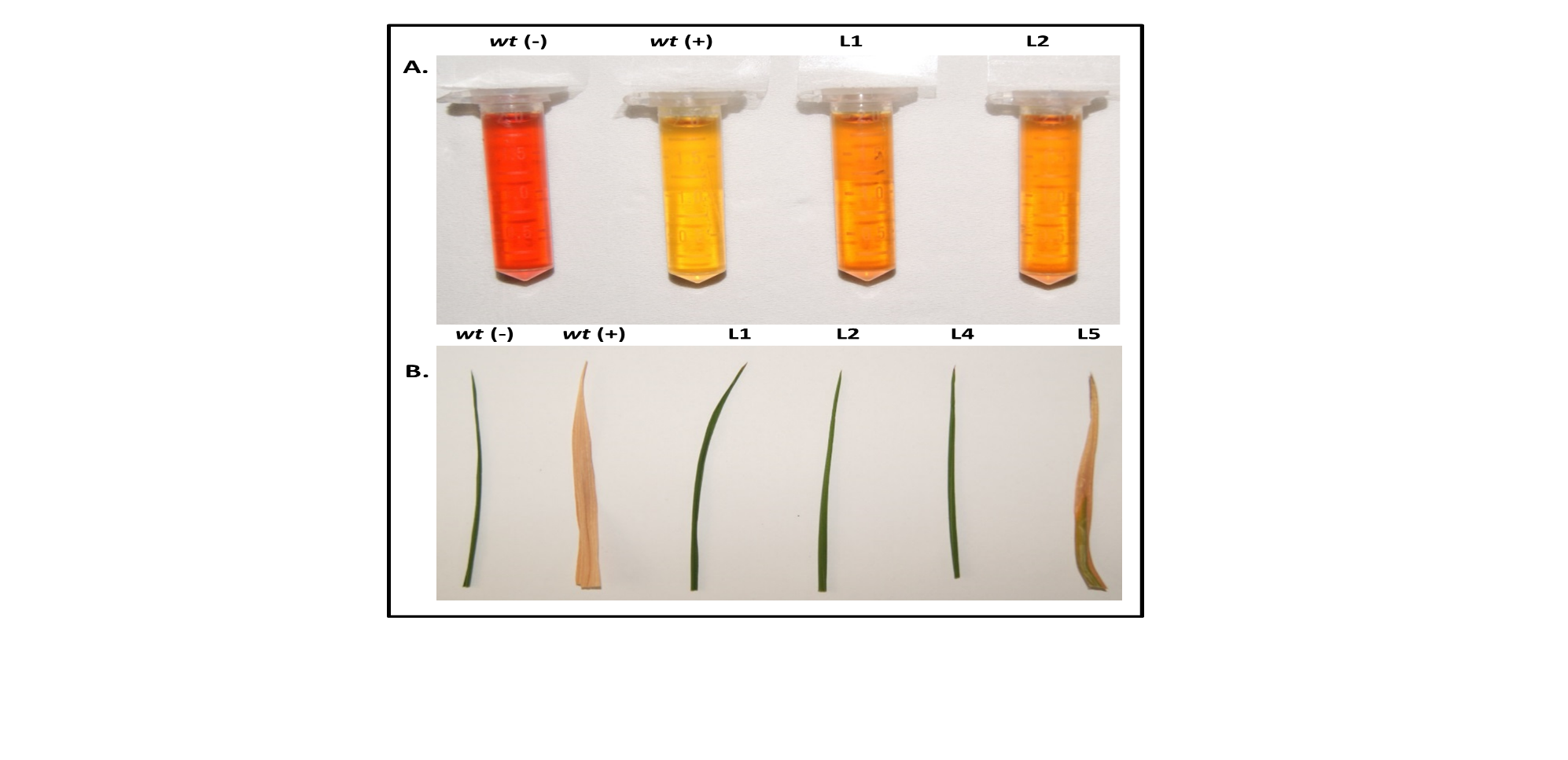


**FIGURE** **SI2.** **Herbicide tolerance assay of transgenic plants. (A)** AHAS color test to confirm the activity of OsmAHAS in transgenic line 1 and 2 in presence of 0.1 μM BM herbicide. **(B)** Leaf paint assay, to analyze the activity of bar in transgenic lines against 3% basta herbicide. Three transgenic lines were tolerant to this herbicide concentration while the line 5 showed susceptibility towards this concentration.


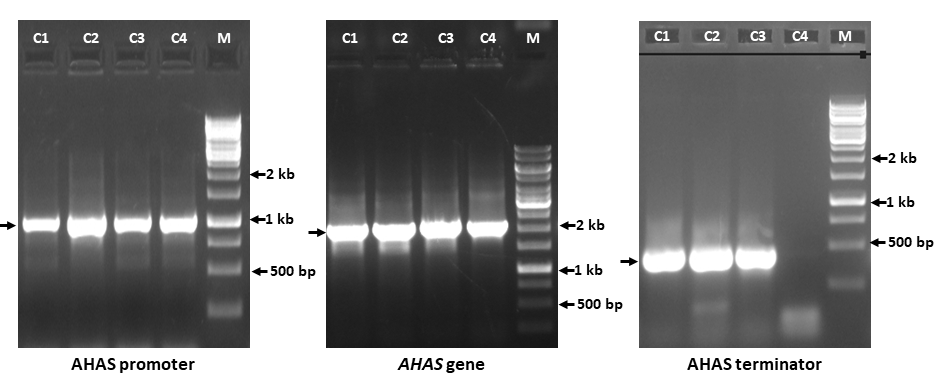


**FIGURE** **SI3.** Full image showing the PCR confirmation of *AHAS* expression cassette.


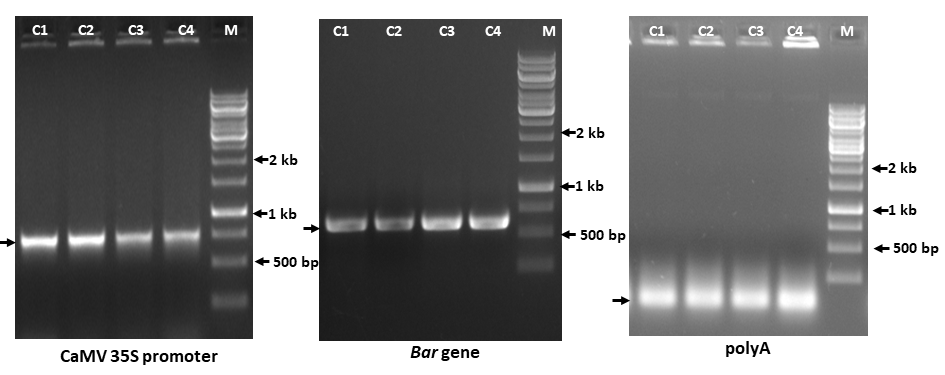


**FIGURE** **SI4.** Full image showing the PCR confirmation of *bar* expression cassette.


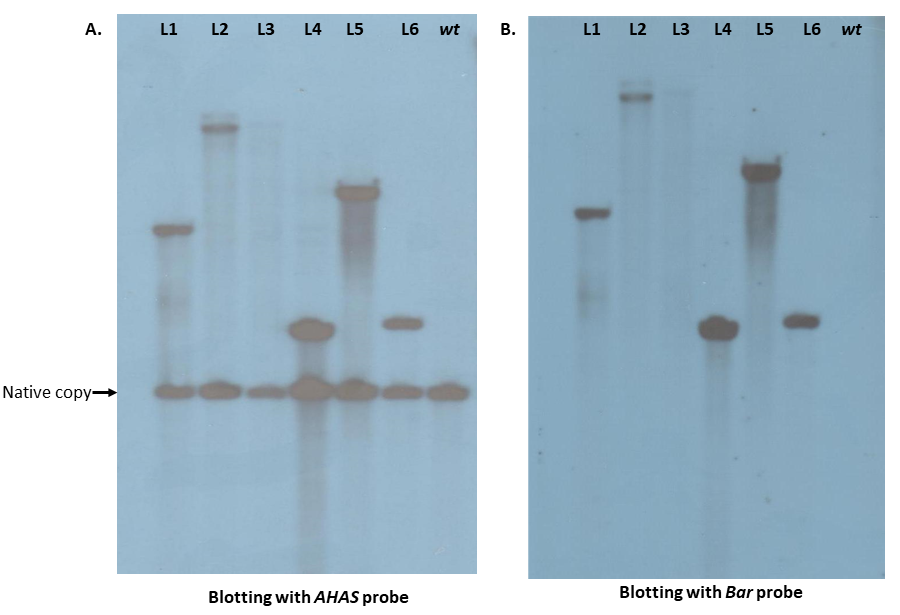


**FIGURE** **SI5.** Full image of southern blotting showing the transgene integration of *AHAS* (A) and *bar* (B) genes. The arrow is indicating native *AHAS* gene in both *wt* and transgenic rice lines.


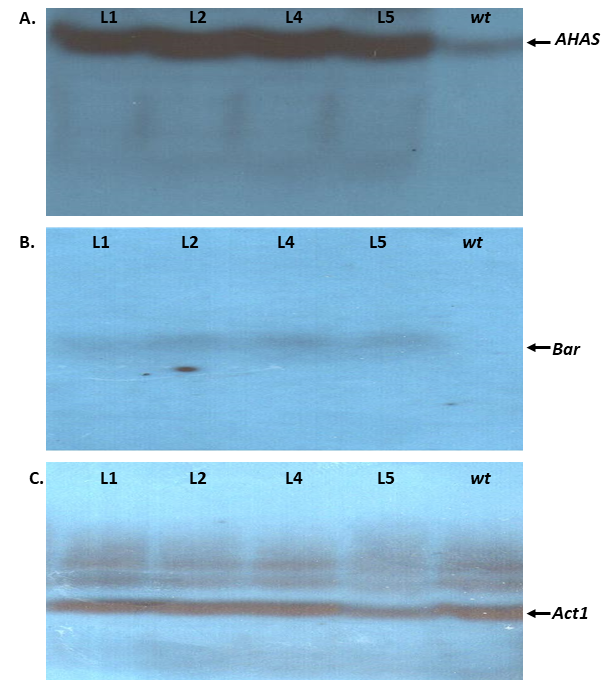


**FIGURE** **SI6.** Northern blotting showing the expression of *AHAS* (A), *bar* (B) and *Act1* (C) genes in *wt* and transgenic lines.


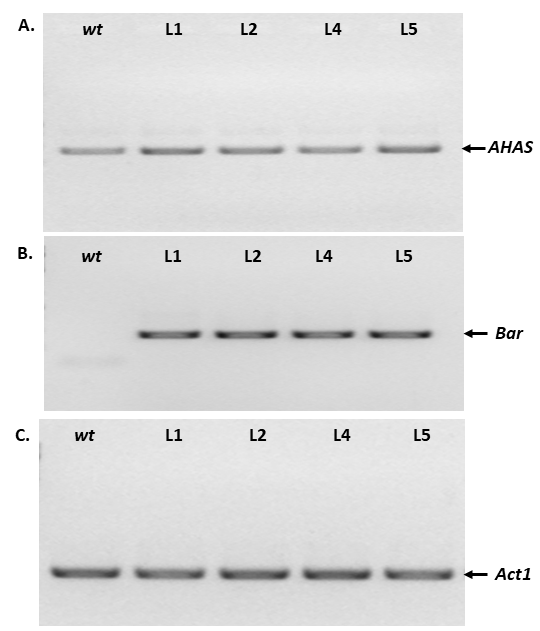


**FIGURE** **SI7.** Semi-quantitative RT-PCR showing the relative expression of *AHAS* (A), *bar* (B) and *Act1* (C) genes in *wt* and transgenic lines.


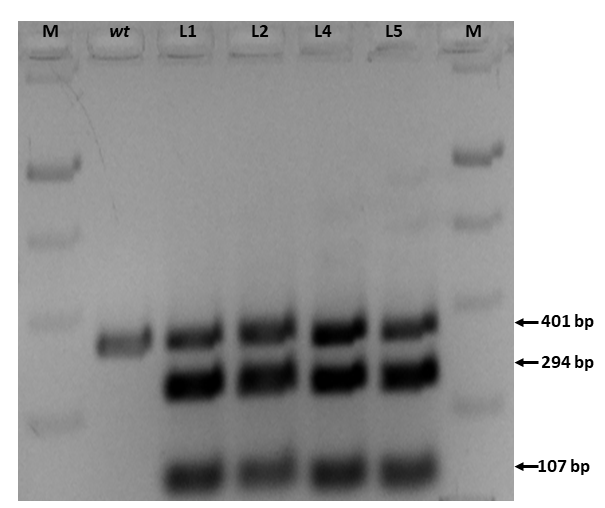


**FIGURE** **SI8.** Restriction digestion analysis of cDNA-amplified PCR products showing the difference between the expression of native and trans-*AHAS* genes in *wt* and transgenic lines.
